# Prevalence, correlates, and associated psychological problems of substance use in Korean adolescents

**DOI:** 10.1186/s12889-016-2731-8

**Published:** 2016-01-27

**Authors:** Subin Park, Yeni Kim

**Affiliations:** 1 Department of Mental Health Research, Seoul National Hospital, 398, Neungdong-ro, Gwangin-gu, Seoul 143-711 South Korea; 2 Department of Child and Adolescent Psychiatry, Seoul National Hospital, 398, Neungdong-ro, Gwangin-gu, Seoul 143-711 South Korea

**Keywords:** Drug use, Risk factor, KYRBS, Adolescents

## Abstract

**Background:**

Substance use among Korean adolescents has been increasing, but little is known about the correlates of substance use in this population. Identification of the correlates is required for development of preventive approaches that aim to reduce or eliminate risk. Therefore, we examined the prevalence and correlates of substance use including psychological problems in a nationwide sample of Korean adolescents.

**Methods:**

Data from the 2014 Korean Youth Risk Behavior Web-Based Survey, collected from 72,060 adolescents aged 12–18 years (mean age 14.94 ± 1.75 years), were analyzed. Participants’ lifetime experiences with substances (alcohol, tobacco, and illicit drugs) were assessed. Participants’ perceived stress, depressive mood, and suicidality during the previous 12 months were also investigated.

**Results:**

The lifetime prevalence estimates of alcohol, tobacco, and illicit drug use were 43.0, 19.9, and 0.4 % of the participants, respectively. The most commonly used illicit drugs were inhalants. Older age, male gender, non-residence with family, low parental educational level and socio-economic status, and low academic achievement were positively and significantly associated with substance use. Substance (alcohol, tobacco, and illicit drug) use was positively and significantly associated with severe stress, depressive mood, and suicidality during the previous 12 months, with the highest odds ratios obtained from illicit drug use.

**Conclusions:**

These results indicate that the use of substances (alcohol, tobacco, and illicit drugs) among Korean adolescents is associated with socially disadvantaged families, psychological problems, and risky behavior. Health education including dependency prevention programs is needed for these high-risk groups.

## Background

Adolescence is an important developmental stage during which new activities, including social, emotional, and sexual relationships, are experienced, and many lifestyle habits are established. Unhealthy behaviors such as drinking, smoking, and illicit drug use commence more commonly during adolescence than at other developmental stages [1]. These behaviors are closely related to increased morbidity and mortality [2, 3]. Teenage drug use is tightly associated with severe substance use problems, psychiatric disorders, and criminal behavior in young adulthood [4–6].

Protective factors against substance use include greater potential for resilience [7, 8], religiosity [9, 10], and effective parental monitoring [11–14]. Risk factors for drug experience include drug use by parents and friends [15, 16], low academic performance [17, 18], low self-esteem [19], depressive symptoms [20], history of stressful events [21], and early use of alcohol and cigarettes [22, 23].

Mental health is another important factor. Studies of data collected from the Global School-Based Health Survey have examined the association between risk behaviors (alcohol, tobacco, and drug use) and mental health, feelings of solitude, social isolation, and insomnia, and have found a significant association between mental health and substance use among adolescents [24]. This association between psychiatric problems and substance use may be bidirectional; psychoactive substances may be used as “self-medication” by patients with psychiatric disorders. For example, alcohol may be used to induce sleep and temporarily alleviate feelings of anxiety and depression [25]. A study of 1285 subjects aged 9 to 18 years found that regular cigarette smoking, alcohol consumption, and illicit substance use in the previous year were each independently associated with an elevated likelihood of diagnosis with psychiatric disorders (i.e. anxiety, mood, or disruptive behavior disorders) [26]. Bipolar disorder and depression are important risk factors, and they often precede substance use in childhood and adolescence [27, 28]. In contrast, substance use is associated with increased risk of psychopathology in adolescents and young adults. The longitudinal Mater-University of Queensland Study of Pregnancy (MUSP) found that adolescent drinking predicted poly-substance use and mental health disorders in young adulthood [29]. An Australian review found that frequency of cannabis use is positively associated with severity of anxiety disorders and depressive symptoms in young people [30].

Historically, available data have shown that illicit drug use is substantially lower in South Korea than in many other countries including the United States and Japan [31]. However, since the 1990s, drug abuse has expanded in Korean society. Students studying abroad have played a key role in expanding the variety of available drugs, from LSD, Ecstasy, and other new drugs, to potent analgesics [32]. According to the data collected by law enforcement agencies, there was an increase in the number of teenage offenders who violated the Harmful Chemical Law from 476 in 2008 to 1127 in 2012. Because of the rapid increase in illicit drug use among Korean adolescents, there is a need for research to identify the correlates of drug use, which are not well-known in this population.

Development of preventive approaches that aim to reduce or eliminate risk requires identification of substance use risk factors. In this study, we investigated prevalence as well as the socio-demographic correlates and associated psychological problems of substance (alcohol, tobacco, and illicit drug) use in a nationally representative sample of Korean adolescents.

## Methods

### Subjects

Data from the 2014 Korean Youth Risk Behavior Web-Based Survey (KYRBS) were used. The KYRBS is a government-approved statistical survey that has been performed annually by the Korea Centers for Disease Control and Prevention since 2005 to monitor health-related risk behaviors among Korean adolescents. A stratified multistage cluster sampling design was used to obtain a nationally representative sample of middle- and high-school students. After the survey was fully explained, only participants who provided informed consent completed the anonymous, self-administered web-based survey during a regular class period. The KYRBS did not collect any identifiers, such as students’ names, schools, telephone numbers, home addresses, or social security numbers. A total of 74,167 students from 400 middle schools and 400 high schools participated in the survey, representing a response rate of 97.2 %. The final sample included 72,060 students (36,470 boys and 35,590 girls, mean age 14.94 ± 1.75 years, range 12–18 years). Additional details about the sampling methodology and survey procedure are available elsewhere [33]. The KYRBS was reviewed and approved by the institutional review board of Korea Centers for Disease Control and Prevention (2014-06EXP-02-P-A). The KYRBS data used for the present study is openly available.

### Measurements

Socio-demographic variables included sex, age, place of residence (name of city), parents’ level of education, residential type, perceived family economic status, and perceived academic achievement. Places of residence were classified as rural area, small city, or large city. Perceived family economic status and academic achievement were assessed by a 5-point Likert scale (low, low-middle, middle, high-middle, and high).

Lifetime illicit drug use was assessed with the following question: “Have you ever used drugs that are often used nonmedically (e.g., inhalants, glue, stimulants, cannabis, amphetamines, marijuana, codeine, neuroleptics) for mood elevation, hallucinatory experience, or excessive dieting?” Participants who responded “Yes” to this question were asked to choose the drugs they had ever used from a list of 33 commonly used illegal drugs classified into nine categories (for example: inhalants, e.g. butane gas, bond, thinner, varnish, lighter gas; stimulants, e.g. caffeine pills; tranquilizers, e.g. Valium, Ativan, Xanax; a large dose of antitussive, e.g. Luminar, Lubiking, Soma, Zinolta; diet pills, e.g. Lasix, Prion; marijuana; Hiropon; other hallucinogens, e.g. ketamine, LSD, Ecstasy, 5-MeO-DiPT, Kratom, Ice; opioid drugs, e.g. opiates, morphine, Demerol, Nubaine; cocaine). The 33 drugs listed were accompanied by more common “street” names or brand names. They were also asked to choose the drug they used the first time.

Lifetime smoking was assessed with the following question: “Have you ever smoked even a puff of a cigarette, cigar, or pipe?” Lifetime alcohol use was assessed with the following question: “Have you ever used alcohol?”

Problematic alcohol use during the last 12 months was assessed with the CRAFFT, a 6-item instrument that is used to screen for alcohol use in the adolescent population. It consists of the following yes-no questions: (1) Have you ever ridden in a Car driven by someone (including yourself) who had been using alcohol? (2) Do you ever use alcohol to Relax, feel better about yourself, or fit in? (3) Do you ever use alcohol while you are by yourself or Alone? (4) Do you ever Forget things you did while using alcohol? (5) Do your Family or Friends ever tell you that you should cut down on your drinking? and (6) Have you ever gotten into Trouble while you were using alcohol? Two or more positive responses indicate the potential for a significant alcohol problem.

The level of perceived stress was measured with the following question: “How much stress do you usually feel?” The response options were very little (1), a little (2), an average amount (3), a lot (4), and very much (5). On the basis of the responses, the participants were classified into the following two groups for multivariate logistic regression analyses: (i) ≤ average perceived stress (1–3) and (ii) > average perceived stress (4–5).

Having experienced a depressive mood during the last 12 months was measured with the following question: “In the past 12 months, have you ever felt depression or hopelessness severe enough to compromise your daily activities during 2 weeks or more?” The response options were “yes” or “no”.

Suicidal ideation was assessed with the question: “Have you seriously thought of committing suicide during the past 12 months?” Existence of a suicide plan was assessed with the question: “Have you concretely planned suicide during the past 12 months?” Suicide attempts were assessed with the question: “Have you attempted suicide during the past 12 months?”

Lifetime sexual relations were assessed with the following questions: “Have you ever experienced sexual relations with a partner of the opposite sex?” and “Have you ever experienced sexual relations with a same-sex partner?”

### Statistical analysis

Descriptive statistics were used to present the prevalence of substance use. Logistic regression tests were performed to compare the socio-demographic factors between substance users and non-users. Odds ratios (ORs) and 95 % confidence intervals (CIs) were calculated using lifetime alcohol, tobacco, and illicit drug use as the main outcome variable and each socio-demographic variable as the principal predictor. To analyze the association between the adolescents’ substance use and psychological variables, logistic regression tests were performed using lifetime alcohol, tobacco, and illicit drug use as the principal predictors and each psychological variable as the main outcome variables, after controlling for age, sex, area of residence, parental education, residential type, socio-economic status, and level of academic achievement. All analyses took into account the sampling design parameters, weighting, clustering, and stratification factor. The proportion of general subject characteristics was weighted according to the respondent’s probability of being selected for the sex-, grade-, and school type-specific distributions for the region [33]. The finite population correction (fpc) factor was used to avoid the overestimation, when developing variance estimates for population parameters. SAS 9.3 (SAS Institute, Cary, NC) was used to perform all other statistical analyses., and a *p*-value less than 0.05 was considered significant.

## Results

Of 72,060 respondents, 30,566 (43.0 %) reported alcohol use (48.4 % of males and 37.0 % of females), 13,967 (19.9 %) reported tobacco use (28.4%of males and 10.6 % of females), and 311 (0.4 %) reported use of any type of illicit drug (0.6 % of males and 0.3 % of females). The most commonly used drug class was inhalant (*n* = 268, 0.37 %), followed by stimulant (*n* = 167, 0.23 %), cannabis (*n* = 161, 0.22 %), and tranquilizer (*n* = 158, 0.22 %). The most common drug class used for the first time was inhalant (*n* = 139, 44.8 % of respondents), followed by cannabis (*n* = 32, 10.3 % of respondents) (Table 1).

**Table 1:** Types of drugs used in Korean adolescents

	Ever used, n (%)	Used the first time, n (% among 196 respondents)
Inhalant	287 (0.4)	139 (44.8)
Stimulant	186 (0.3)	24 (7.7)
Tranquilizer	177 (0.2)	27 (8.7)
Large dose of antitussive	170 (0.2)	21 (6.8)
Diet pill	154 (0.2)	15 (4.8)
cannabis	161 (0.2)	32 (10.3)
Amphetamine	156 (0.2)	15 (4.8)
Other hallucinogen	153 (0.2)	11 (3.5)
Opioid drug	152 (0.2)	20 (6.5)
Cocaine	141 (0.2)	6 (1.9)

Older age (OR = 1.37 for an increase of 1 year), male gender (OR = 1.71 compared to female gender), non-residence with family (ORs = 18.03 for residence with relatives, 6.70 for residence with friends, alone, or in a dormitory, and 88.78 for residence in a facility, compared with residence with family), and alcohol and tobacco use (ORs = 7.64 and 15.01) were positively and significantly associated with illicit drug use. High paternal and maternal educational levels (ORs = 0.30 and 0.24 for college degree or higher, and 0.28 and 0.17 for high school education, compared with middle school education or lower), socio-economic status (ORs = 0.42 for high level, and 0.06 for middle level, compared with low level), and academic achievement (ORs = 0.67 for high level, 0.24 for middle level, compared with low level) were negatively and significantly associated with illicit drug use (Table 2).

**Table 2:** Socio-demographic characteristics of substance users and non-users among Korean adolescents

	Substance users (*n* = 311), %	Non-users (*n* = 71,749), %	OR (95 % CI)	*p*-value
Age (years), mean (95 % CI)	15.90 (15.70–16.10)	15.00 (14.96–15.04)	1.37 (1.27–1.48)	<0.001
Sex, male, %	65.1	52.1	1.71 (1.33–2.21)	<0.001
Area of residence, %				
Rural	8.2	6.5	ref	
Small city	46.0	49.9	0.83 (0.53–1.32)	0.441
Large city	45.8	43.6	0.73 (0.46–1.16)	0.184
Paternal Education, %				
Middle school education or lower	11.0	3.4	ref	
High school education	32.4	36.5	0.28 (0.17–0.46)	<0.001
College degree or higher	56.6	60.0	0.3 (0.19–0.47)	<0.001
Maternal Education, %				
Middle school education or lower	12.8	3.0	ref	
High school education	35.1	46.9	0.17 (0.1–0.29)	<0.001
College degree or higher	52.1	50.2	0.24 (0.15–0.39)	<0.001
Residential type, %				
With family	59.5	96.1	ref	
With relatives	9.0	0.8	18.03 (12.38–26.25)	<0.001
With friends, alone, domitory	11.2	2.7	6.7 (4.61–9.73)	<0.001
Facility	20.3	0.4	88.78 (63.81–123.54)	<0.001
Socio-economic status, %				
Low	25.0	3.7	ref	
Low-Middle	16.4	14.2	0.17 (0.12–0.24)	<0.001
Middle	19.9	48.4	0.06 (0.04–0.09)	<0.001
High-Middle	16.4	25.7	0.1 (0.06–0.15)	<0.001
High	22.3	7.9	0.42 (0.31–0.57)	<0.001
Academic achievement, %				
Low	29.9	10.8	ref	
Low-Middle	18.9	24.1	0.29 (0.2–0.4)	<0.001
Middle	18.4	28.0	0.24 (0.17–0.33)	<0.001
High-Middle	10.2	24.9	0.15 (0.1–0.23)	<0.001
High	22.6	12.2	0.67 (0.5–0.9)	0.008
Alcohol use	85.1	42.8	7.64 (5.5–10.62)	<0.001
Tobacco use	78.6	19.7	15.01 (11.41–19.75)	<0.001

*OR* odd ratios, *CI* confidence intervals

Older age (OR = 1.35 for an increase of 1 year), male gender (OR = 3.33 compared to female gender), non-residence with family (ORs = 2.37 for residence with relatives, 1.24 for residence with friends, alone, or in a dormitory, and 3.32 for residence in a facility, compared with residence with family), and alcohol use (OR = 9.22) were positively and significantly associated with tobacco use. High paternal and maternal educational levels (ORs = 0.45 and 0.47 for college degree or higher, and 0.67 and 0.69 for high school education, compared with middle school education or lower), socio-economic status (ORs = 0.37 for high level and 0.41 for middle level, compared with low level), and academic achievement (ORs = 0.27 for high level and 0.37 for middle level, compared with low level) were negatively and significantly associated with drug use (Table 3).

**Table 3:** Socio-demographic characteristics of tobacco users and non-users among Korean adolescents

	Tobacco users (*n* = 13,967), %	Non-users (*n* = 58,094), %	OR (95 % CI)	*p*-value
Age (years), mean (95 % CI)	15.70 (1.54)	14.83 (1.75)	1.35 (1.34–1.35)	<0.001
Sex, male, %	74.4	46.6	3.33 (3.31–3.25)	<0.001
Area of residence, %				
Rural	7.4	6.3	ref	
Small city	50.3	49.8	0.86 (0.86–0.87)	<0.001
Large city	42.4	43.9	0.83 (0.82–0.84)	<0.001
Paternal Education, %				
Middle school education or lower	5.5	3.0	ref	
High school education	43.3	34.8	0.67 (0.66–0.68)	<0.001
College degree or higher	51.2	62.2	0.45 (0.44–0.45)	<0.001
Maternal Education, %				
Middle school education or lower	4.5	2.6	ref	
High school education	53.4	45.2	0.69 (0.68–0.70)	<0.001
College degree or higher	42.1	52.1	0.47 (0.46–0.48)	<0.001
Residential type, %				
With family	94.2	96.4	ref	
With relatives	1.6	0.7	2.37 (2.31–2.43)	<0.001
With friends, alone, domitory	3.2	2.6	1.24 (1.22–1.26)	<0.001
Facility	1.0	0.3	3.32 (3.22–3.43)	<0.001
Socio–economic status, %				
Low	7.1	3.0	ref	
Low-Middle	18.8	13.1	0.62 (0.61–0.62)	<0.001
Middle	46.1	48.8	0.41 (0.40–0.41)	<0.001
High-Middle	21.0	26.8	0.34 (0.33–0.34)	<0.001
High	7.0	8.2	0.37 (0.37–0.38)	<0.001
Academic achievement, %				
Low	20.1	8.7	ref	
Low-Middle	28.8	22.9	0.54 (0.54–0.55)	<0.001
Middle	24.7	28.8	0.37 (0.36–0.37)	<0.001
High-Middle	18.0	26,5	0.29 (0.29–0.30)	<0.001
High	8.4	13.2	0.27 (0.27–0.28)	<0.001
Alcohol use	82.1	33.2	9.22 (9.16–9.28)	<0.001

*OR* odd ratios, *CI* confidence intervals

Older age (OR = 1.44 for an increase of one year), male gender (OR = 1.60 compared to female gender), and non-residence with family (ORs = 1.65 for residence with relatives, 2.05 for residence with friends, alone, or in a dormitory, and 1.91 for residence in a facility, compared with residence with family) were positively and significantly associated with alcohol use. High paternal and maternal educational levels (ORs = 0.48 and 0.53 for college degree or higher, and 0.71 and 0.79 for high school education, compared with middle school education or lower), socio-economic status (ORs = 0.42 for high level and 0.54 for middle level, compared with low level), and academic achievement (ORs = 0.41 for high level and 0.55 for middle level, compared with low level) were negatively and significantly associated with drug use (Table 4).

**Table 4:** Socio-demographic characteristics of alcohol users and non-users among Korean adolescents

	Alcohol users (*n* = 30,566), %	Non-users (*n* = 41,494), %	OR (95 % CI)	*p*-value
Age (years), mean (95 % CI)	15.60 (1.65)	14.56 (1.68)	1.44 (1.43–1.44)	<0.001
Sex, male, %	58.8	47.2	1.60 (1.59–1.61)	<0.001
Area of residence, %				
Rural	7.5	5.7	ref	
Small city	49.7	50.1	0.76 (0.75–0.77)	<0.001
Large city	42.8	44.2	0.74 (0.73–0.75)	<0.001
Paternal Education, %				
Middle school education or lower	4.6	2.6	ref	
High school education	41.1	33.0	0.71 (0.70–0.72)	<0.001
College degree or higher	54.4	64.4	0.48 (0.48–0.49)	<0.001
Maternal Education, %				
Middle school education or lower	3.7	2.4	ref	
High school education	52.0	42.8	0.79 (0.78–0.80)	<0.001
College degree or higher	44.2	54.7	0.53 (0.52–0.53)	<0.001
Residential type, %				
With family	94.7	96.9	ref	
With relatives	1.2	0.6	2.05 (2.00–2.10)	<0.001
With friends, alone, domitory	3.5	2.2	1.65 (1.63–1.67)	<0.001
Facility	0.6	0.3	1.91 (1.85–1.97)	<0.001
Socio-economic status, %				
Low	5.2	2.8	ref	
Low-Middle	17.1	12.1	0.77 (0.76–0.78)	<0.001
Middle	47.8	48.6	0.54 (0.53–0.54)	<0.001
High-Middle	23.0	27.7	0.46 (0.45–0.46)	<0.001
High	6.8	8.8	0.42 (0.42–0.43)	<0.001
Academic achievement, %				
Low	14.3	8.4	ref	
Low-Middle	26.5	22.2	0.70 (0.70–0.71)	<0.001
Middle	27.0	28.7	0.55 (0.55–0.56)	<0.001
High-Middle	22.4	26.6	0.49 (0.49–0.50)	<0.001
High	9.8	14.1	0.41 (0.40–0.41)	<0.001

*OR* odd ratios, *CI* confidence intervals

After adjusting for age, sex, area of residence, parental education, residential type, socio-economic status, and academic achievement, illicit drug users were more likely to experience severe stress (OR = 1.98), depressive mood (OR = 3.34), suicidal ideation (OR = 4.52), suicide plan (OR = 9.93), suicide attempt (OR = 13.13), and problematic drinking (OR = 12.90) during the past 12 months, as well as sexual relations with the opposite sex (OR = 9.92) and with a same-sex partner (OR = 28.27), compared to non-users. Tobacco users were more likely to experience severe stress (OR = 1.32), depressive mood (OR = 1.75), suicidal ideation (OR = 1.83), suicide plan (OR = 1.97), suicide attempt (OR = 2.91), and problematic drinking (OR = 10.90) during the past 12 months, as well as sexual relations with the opposite sex (OR = 5.15) and with a same-sex partner (OR = 3.02), compared to non-users. Alcohol users were more likely to experience severe stress (OR = 1.33), depressive mood (OR = 1.71), suicidal ideation (OR = 1.66), suicide plan (OR = 1.87), and suicide attempt (OR = 2.26) during the past 12 months, as well as sexual relations with the opposite sex (OR = 3.23) and with a same-sex partner (OR = 1.93), compared to non-users (Table 5).

**Table 5:** Associations between substance (drug, tobacco, alcohol) use and psychological and behavioral problems

	Illicit drug users (*N* = 311), %	Non-users (*N* = 71,749), %	AOR (95 % CI)	*p*-value	Tobacco users (*n* = 13,967), %	Non-users (*n* = 58,094), %	AOR (95 % CI)	*p*-value	Alcohol users (*n* = 30,566), %	Non-users (*n* = 41,494), %	AOR (95 % CI)	*p*-value
Perceived stress	48.2	36.9	1.98 (1.88–2.08)	<0.001	42.3	35.7	1.32 (1.31–1.33)	<0.001	41.6	33.5	1.33 (1,32–1.34)	<0.001
Depressive mood	57.6	26.6	3.34 (3.17–3.52)	<0.001	35.7	24.5	1.75 (1.73–1.76)	<0.001	33.2	21.8	1.71 (1.70–1.72)	<0.001
Suicide idea	49.5	12.9	4.52 (4.29–4.76)	<0.001	18.8	11.6	1.83 (1.81–1.84)	<0.001	16.4	10.5	1.66 (1.65–1.67)	<0.001
Suicide plan	41.0	4.3	9.93 (9.39–10.49)	<0.001	7.3	3.7	1.97 (1.94–2.00)	<0.001	5.9	3.3	1.87 (1.84–1.89)	<0.001
Suicide attempt	34.1	2.8	13.13 (12.37–13.93)	<0.001	5.6	2.3	2.91 (2.86–2.96)	<0.001	4.0	2.1	2.26 (2.22–2.30)	<0.001
Problematic drinking	45.2	6.3	12.90 (12.21–13.63)	<0.001	24.2	2.1	10.90 (10.77–11.04)	<0.001	15.1	-		
Sexual relations with the opposite sex	41.5	4.5	9.92 (9.39–10.48)	<0.001	14.3	2.3	5.15 (5.09–5.22)	<0.001	8.3	2.0	3.23 (3.18–3.28)	<0.001
Sexual relations with the same sex	46.9	0.8	28.27 (26.43–30.24)	<0.001	2.7	0.6	3.02 (2.92–3.12)	<0.001	1.5	0.6	1.93 (1.87–2.00)	<0.001

*AOR* odds ratios adjusted for age, sex, area of residence, parental education, residential type, socio-economic status, and academic achievement, *CI* confidence interval

## Discussion

This is the first nationally representative study of adolescent substance use to include illegal drugs as well as alcohol and tobacco in South Korea. Frequencies of alcohol use and smoking among Korean adolescents were similar to those among adolescents in other countries [34–36]. However, frequency of illicit drug use among Korean adolescents was observed to be much lower than among adolescents in other countries [20, 22, 24, 36, 37].

Inhalants and solvents was the most common drug class used for the first time. These substances may be chosen due to their relative availability, low cost, and rapid onset of effect [38]. Individuals using glues or thinners for the purposes of hallucination and stimulation, or selling these substances for these purposes, may be charged under the Harmful Chemical Control Law in South Korea [32]; however, butane gas, adhesives, and glues containing toluene are sold widely and can be purchased easily over the counter. Inhalant use may be followed by other illicit drugs, as adolescents expand the variety of drugs used.

Consistent with previous studies in other countries [39, 40], males and older adolescents more commonly used substances compared to females and younger adolescents. Living with parents has a protective effect against substance use, while low parental education level and low socio-economic status was associated with substance use. These results are consistent with previous studies that have reported the importance of family supervision and living with parents to reduce the likelihood of substance use [11–14].

The association between low academic achievement and substance use may be bidirectional. Students may use substances to cope with their anxiety over academic failure [18, 41]. Moreover, poor academic performers may be more likely to skip school, have disciplinary problems, and engage in deviant behaviors than high achievers, and this may create a social structure that encourages substance use [18, 42]. In contrast, substance use may be a contributory factor to academic difficulties, via skipping class and reduced academic motivation [41, 43]. A third explanation is that substance use and failing academic performance are the result of shared factors, such as a general propensity for deviance [44]. As we did not obtain relevant information about temporality, no inferences about causality are possible in this study.

Previous studies have reported that alcohol, tobacco, and illicit drug use and sexual risk-taking are highly comorbid [45], and that alcohol and tobacco are used first, followed by illicit drugs [23]. Consistently, we found inter-relationships between sexual activity and use of alcohol, tobacco, and illicit drugs.

Associations between substance use and severe stress, depression, and suicidality were consistent with previous studies in other countries [20, 21, 46]. Adolescents may initiate or accelerate substance-use behaviors as a way to cope with their stress, negative mood, and suicidal ideation. Another possible explanation is that their perceived stress and negative mood may be elicited as symptoms of withdrawal from the drugs, which in turn leads to repetitive use of the drugs. Because of such interactive or circular relationships, multifaceted approaches that treat or prevent adolescent depression and/or suicidality, while dissuading adolescent involvement in substance-use behaviors, may both improve their mental health and reduce risky behaviors.

The limitations of this study include that depressive mood and perceived stress was measured using one item each, rather than with standardized multi-item measures. This may limit the validity of measurements of these variables. Additionally, the prevalence of substance use and sexual activity might be underestimated because this survey was conducted in school and did not include adolescents who were not attending school, a high-risk group for delinquent behavior [44, 47]. Moreover, social desirability may have biased the survey results. Although the survey was anonymous, drug use may have been underreported due to reluctance to disclose illegal drug use. Additionally, due to inadequate statistical power, the present study was unable to examine correlates of use of each type of drug. Furthermore, frequency of substance use, and sex risk level (e.g. unprotected sex; sex with multiple partners), were not assessed in this study. Finally, as we did not obtain relevant information about temporality, inference of a causal relationship between substance use, psychological problems, and risky behavior is impossible. However, the strength of the present study is its inclusion of data from 72,060 adolescents from a nationally representative sample in South Korea, which improves external validity of the study results.

## Conclusions

Even within the context of these limitations, the results of the present study indicate that the use of substances (alcohol, tobacco, and illicit drugs) among Korean adolescents is associated with socially disadvantaged families, psychological problems, and risky behavior. Health education including dependency prevention programs is needed for these high risk groups in South Korea. Future prospective studies are required in order to identify casual relationships among substance use, psychopathology, and suicidality.

## Abbreviations

ODD: oppositional defiant disorder
CD: conduct disorder
ADHD: attention deficit hyperactivity disorder
ORs: odds ratios
CIs: confidence intervals
